# Relationship between facial skin problems with a focus on inflammatory cytokines and the presence of *Malassezia* in 1-month-old infants

**DOI:** 10.1038/s41598-023-31949-2

**Published:** 2023-03-28

**Authors:** Satsuki Shimizu, Kaori Yonezawa, Megumi Haruna, Emi Tahara-Sasagawa, Yuriko Usui, Takeo Minematsu, Sachi Higuchi

**Affiliations:** 1 Department of Midwifery and Women’s Health, Division of Health Sciences and Nursing, Graduate School of Medicine, The University of Tokyo, 7-3-1 Hongo, Bunkyo-Ku, Tokyo, 113-0033 Japan; 2 Global Nursing Research Center, Graduate School of Medicine, The University of Tokyo, 7-3-1 Hongo, Bunkyo-Ku, Tokyo, 113-0033 Japan; 3 Department of Skincare Science, Graduate School of Medicine, The University of Tokyo, 7-3-1 Hongo, Bunkyo-Ku, Tokyo, 113-0033 Japan; 4 Ishikawa Prefectural Nursing University, 1-1 Gakuendai, Kahoku-City, Ishikawa 929-1210 Japan; 5 Department of Midwifery, Oita University of Nursing and Health Sciences, 2944-9 Megusuno, Oita-City, Oita 870-1201 Japan

**Keywords:** Immunology, Microbiology, Pathogenesis, Signs and symptoms

## Abstract

Infantile skin problems not only cause temporary pain and discomfort, but also have a long-term impact on health. Hence, the purpose of this cross-sectional study was to clarify the relationship between inflammatory cytokines and *Malassezia* fungal facial skin problems in infants. Ninety-six 1-month-old infants were examined. Facial skin problems and the presence of inflammatory cytokines in the forehead skin were assessed using the infant facial skin visual assessment tool (IFSAT) and the skin blotting method, respectively. *Malassezia,* a fungal commensal, was detected using forehead skin swabs, and its percentage in the total fungal population was analyzed. Infants with positive interleukin-8 signals were more likely to have severe facial skin problems (*p* = 0.006) and forehead papules (*p* = 0.043). No significant association between IFSAT scores and *Malassezia* was found, but infants with forehead dryness had a lower percentage of *M. arunalokei* in the total fungal population (*p* = 0.006). No significant association was observed between inflammatory cytokines and *Malassezia* in the study participants. Longitudinal studies on the development of facial skin problems in infants are warranted to investigate the involvement of interleukin-8 and devise preventive strategies in the future.

## Introduction

The skin of infants is immature and the stratum corneum is thinner than that of adults, resulting in a weak barrier function that imperfectly prevents dryness and the entry of external irritants and allergens^1^. As a result, their skin is more susceptible to the effects of commensal organisms and may be more prone to inflammation than adults. Therefore, skin problems such as erythema, papules, and dryness develop relatively easily. In infants, skin problems cause temporary discomfort, including itching and pain, and can have a long-term impact on health. Skin problems weaken the skin barrier function, making it easier for allergens to enter the body.

Skin problems increase the risk of future allergic diseases, such as atopic dermatitis and food allergies^2,3^. Furthermore, parents may feel anxious or guilty when their infants have skin problems, given that infantile skin problems most commonly occur early in an infant’s life. Sugiyama et al.^4^ revealed that 29.3% of parents reported being concerned about their infant’s skin condition at the 1-month checkup. Yonezawa et al.^5^ showed that infantile skin problems were consequently associated with poor quality of life in mothers.

The face is one of the most common areas to develop skin problems in infancy, including seborrheic dermatitis, neonatal acne, atopic dermatitis, and miliaria. Yonezawa et al.^6^ showed that > 75% of infants developed some facial skin problems by 3 months of age, representing a higher incidence of skin problems in the face than in the diaper area and other body parts, such as the limbs, chest, abdomen, and back. Shiga et al.^7^ previously reported that infantile facial skin problems were most likely to occur at approximately 1 month of age.

Infantile seborrheic dermatitis, neonatal acne, and atopic dermatitis, common infantile facial skin problems, have been suggested to be linked to *Malassezia*, a fungal genus, as the cause or aggravating factor^7–16^. *Malassezia* is a fungal species indigenous to the skin of humans and animals and requires sebum for its growth. Currently, there are 18 known species of *Malassezia*^17^, and 10 of these are human commensal organisms^18,19^. The main *Malassezia* species found in healthy adults and infants are *M*. *restricta* and *M. globosa*, accounting for more than 70–90% of all *Malassezia* species^20,21^.

Previous studies suggest that infantile seborrheic dermatitis was associated with *M. restricta*, *M. globosa*, *M. furfur*, and *M. sympodialis*^11,14–16^. Moreover, neonatal acne was associated with *M. sympodialis*^8^, and atopic dermatitis was associated with *M. restricta*, *M. globosa*, and *M. furfur*^9,10,12,13^. These results suggest that these four *Malassezia* species—*M. restricta*, *M. globosa*, *M. furfur*, and *M. sympodialis*—are particularly influential in infantile facial skin problems. However, in these previous studies, a unified classification and evaluation method for detecting *Malassezia* are lacking. Moreover, the detection rate of *Malassezia* was reported to be relatively low when culture methods were used^22^. Therefore, to examine the involvement of *Malassezia* in infantile facial skin problems, it is necessary to evaluate its presence accurately using genetic testing.

There are two possible mechanisms by which *Malassezia* can cause infantile facial skin problems. The first mechanism is that keratinocytes, which make up most of the epidermis and are responsible for the skin’s natural immunity, recognize *Malassezia* and produce inflammatory cytokines, resulting in the induction of inflammation. The second mechanism is the acquired immunity pathway in the skin. Langerhans cells first recognize *Malassezia* in the epidermis. They then move to the dermis and present the antigens to naive T cells, producing inflammatory cytokines from Th2 and Th17 cells and resulting in the induction of inflammation.

Previous studies investigating the relationship of *M. restricta*, *M. globosa*, *M. furfur,* and *M. sympodialis* with inflammatory cytokines showed that: (1) *M. restricta* increases interleukin (IL)-4 and IL-8 levels^23,24^; (2) *M. globosa* increases IL-5, IL-6, IL-8, IL-10, and IL-23 levels^23,24^; (3) *M. sympodialis* increases IL-1β, IL-6, IL-8, IL-17, and tumor necrosis factor (TNF)-α levels^24–26^; and (4) *M. furfur* increases IL-6, IL-8, IL-17, and TNF-α levels^25,27^. Previous studies have indicated a strong association between *Malassezia* and IL-4, IL-6, and IL-8, while IL-17 is known to be produced by Th17 cells, which are generally believed to be responsive to fungi; therefore, we chose Il-4, IL-6, IL-8, and IL-17 as evaluation indicators in this study. However, these aforementioned studies were conducted using human T-cells or normal human epidermal keratinocytes^23–27^. To the best of our knowledge, no study has investigated the relationship between the presence of *Malassezia* and inflammatory cytokines in infants.

Skin blotting is one method used to collect inflammatory cytokines in the epidermis and dermis of the skin. Previous studies have shown that skin blotting can detect soluble molecules in the deep dermis and epidermis, and that the intensity of detection correlates with the concentration of inflammatory cytokines within the skin^28,29^. Therefore, it has been recognized as an alternative method to skin biopsy. Skin blotting is a recently developed method of assessing skin conditions. It can non-invasively extract soluble molecules leaking from inside the skin by attaching a nitrocellulose membrane to the skin’s surface^28^. Skin blotting is a safe technique that is used not only in adults but also in newborns^30^. In adults, Tamai et al.^31^ reported a correlation between the intensity of albumin detected by skin blotting and transepidermal water loss (TEWL). TEWL is a standard measure of skin barrier function, and high TEWL levels indicate a lowered skin barrier function. Furthermore, Higuchi et al.^30^ reported significantly increased IL-6 and TNF-α levels in a skin rash area among early neonates compared to those in a non-rash area. If skin blotting reveals the presence of inflammatory cytokines associated with infantile facial skin problems, it may contribute to our understanding of the pathogenesis of infantile facial skin problems.

**Figure 1:**
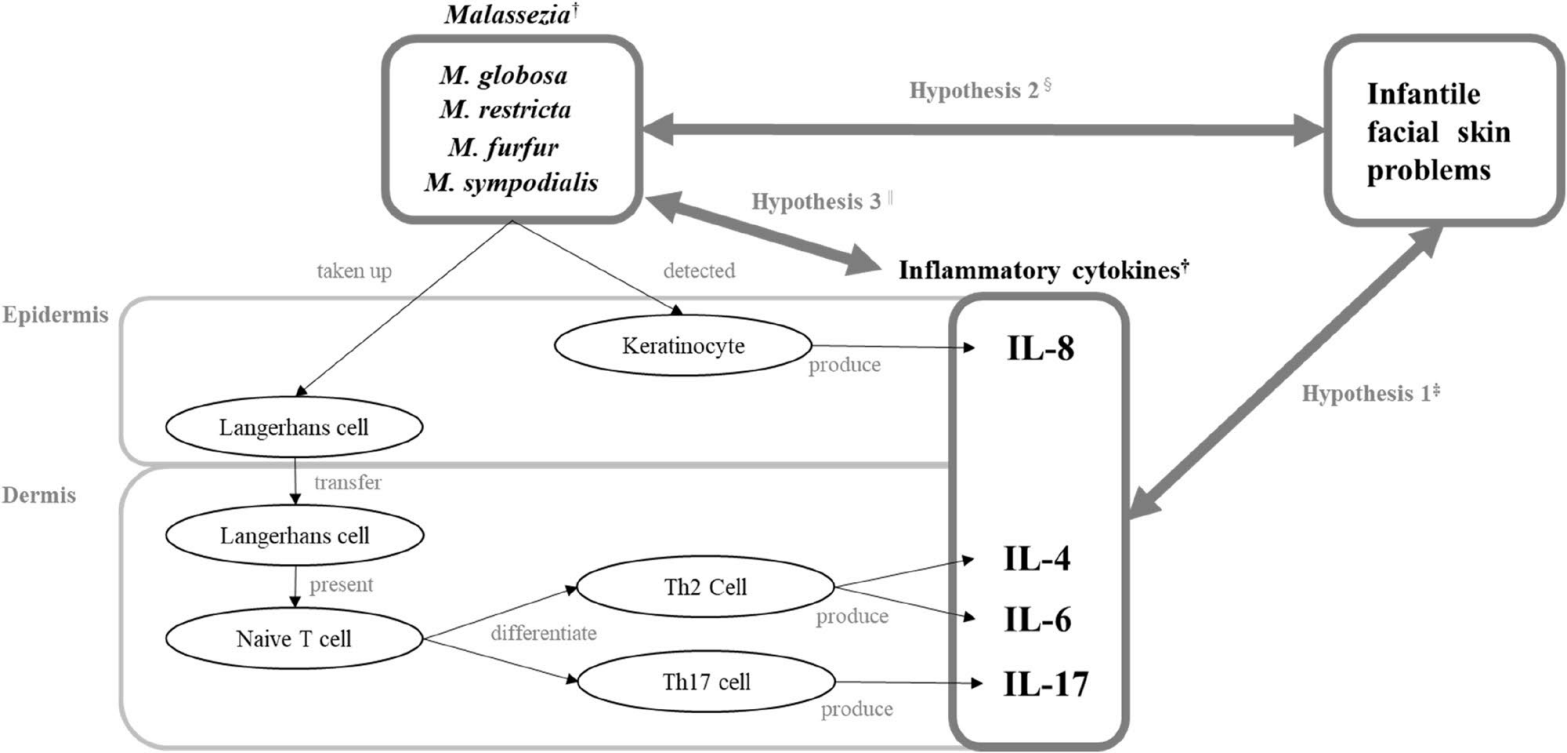
Model for hypotheses. ^†^*Malassezia* and inflammatory cytokines were obtained from the forehead. ^‡^Hypothesis 1: Inflammatory cytokines are involved in facial skin problems in infants. ^§^Hypothesis 2: *Malassezia* is involved in facial skin problems in infants. ^||^Hypothesis 3: Inflammatory cytokines and *Malassezia*, both of which are involved in facial skin problems in infants, are associated with each other.

Therefore, this study aimed to identify the association between inflammatory cytokines and *Malassezia* presence for facial skin problems in 1-month-old infants. There were three hypotheses for this study. First, inflammatory cytokines are involved in infantile facial skin problems. Second, *Malassezia* is involved in infantile facial skin problems. Third, inflammatory cytokines and *Malassezia*, both of which are involved in infantile facial skin problems, are associated with each other. If this study successfully clarifies the relationship between inflammatory cytokines and *Malassezia* in facial skin problems among 1-month-old infants, it may shed light on the pathogenic mechanism underlying infantile facial skin problems and lead to the development of possible preventive strategies for these skin problems (Fig. 1).

## Results

### Participant selection

A total of 121 families (comprising infants and their parents) agreed to participate in this study as follows: (1) 23 families were recruited via the hospital (participation rate, 71.9%); (2) 91 families were recruited via the snowball sampling method (participation rate, 96.8%); and (3) seven families were recruited via flyers, posters, and the laboratory website (participation rate, 100.0%).

Of the 121 infants who participated in the study, only 96 were analyzed for the presence of *Malassezia* because of cost limitations. Therefore, 96 infants with data on *Malassezia* were included in the analysis. Data was collected from 96 infants in the hospital examination room (*n* = 23 [24.0%]), or the home of the participant (*n* = 67 [69.8%]), or in a laboratory room at the university (*n* = 6 [6.3%]). The median (interquartile range) infant age (in days) at the time of data collection was 32 (30–39) days.

### Infantile facial skin problems

The median (interquartile range) infant facial skin visual assessment tool (IFSAT) score was 8.0 (4.0–12.0). Overall, 55 infants (57.3%) had severe facial skin problems with a score of 8 points or more; 16 infants (16.7%) had forehead erythema; 46 infants (47.9%) had forehead papules; and seven infants (7.3%) had forehead dryness. As for forehead exudates and yellow scaling, two infants (2.1%) had symptoms, but due to the small sample size, the relationship between exudates and yellow scaling could not be analyzed. Only six infants (6.3%) had no facial skin problems, with an IFSAT score of zero.

The IFSAT score was extracted only for the forehead item and was analyzed for correlation with the face score. The forehead and face scores were positively correlated (*ρ* = 0.826, *p* < 0.001).

### Participant characteristics

Table 1 summarizes the characteristics of the participants and infantile forehead skin barrier function and clarifies the relationship between each characteristic and the following infantile facial skin problems: the IFSAT scores, severe facial skin problems, forehead erythema, forehead papules, and forehead dryness.

**Table 1:** Relationship of participants’ characteristics with IFSAT scores, severe facial skin problems, forehead erythema, forehead papules, and forehead dryness (n = 96).

	Total (n = 96)		IFSAT^†^ scores (n = 96)				Severe facial skin problems^‡^ (n = 55, 57.3%)			Forehead erythema (n = 16, 16.7%)			Forehead papules (n = 46, 47.9%)			Forehead dryness (n = 7, 7.3%)		
	n (%) or median (IQR^§^)		Median (IQR^§^)		*ρ*	*p*-value	n (%) or median (IQR^§^)		*p*-value	n (%) or median (IQR^§^)		*p*-value	n (%) or median (IQR^§^)		*p*-value	n (%) or median (IQR^§^)		*p*-value
Infants’ sex						0.991^||^			0.650^¶^			0.139^¶^			0.447^¶^			0.097^¶^
Male	40	(41.7)	8.0	(3.0–12.0)			24	(60.0)		4	(10.0)		21	(52.5)		5	(12.5)	
Female	56	(58.3)	8.0	(4.0–12.5)			31	(55.4)		12	(21.4)		25	(44.6)		2	(3.6)	
Gestational age (weeks)	39.0	(38.0–40.0)			− 0.095	0.359^††^	39.0	(38.0–40.0)	0.268^||^	38.0	(38.0–39.8)	0.352^||^	39.0	(38.0–40.0)	0.127^||^	39.0	(38.0–39.0)	0.555^||^
Mode of delivery						0.419^||^			0.081^¶^			0.474^¶^			0.267^¶^			**0.007**^‡‡^
Vaginal birth	85	(88.5)	8.0	(4.0–12.5)			46	(54.1)		15	(17.6)		39	(45.9)		4	(4.7)	
Cesarean section	11	(11.5)	8.0	(8.0–11.0)			9	(81.8)		1	(9.1)		7	(63.6)		3	(27.3)	
Mothers’ parity						0.851^||^			0.702^¶^			0.523^¶^		(63.6)	0.831^¶^			0.057^¶^
Primipara	49	(51.0)	8.0	(5.0–11.5)			29	(59.2)		7	(14.3)		24	(49.0)		6	(12.2)	
Multipara	47	(49.0)	8.0	(3.0–13.0)			26	(55.3)		9	(19.1)		22	(46.8)		1	(2.1)	
Birth weight (g)	3021	(2783–3230)			0.087	0.397^††^	3064	(2820–3334)	0.422^||^	2940	(2786–3190)	0.810^||^	3069	(2820–3359)	0.221^||^	2736	(2642–2975)	**0.049**^||^
Birth height (cm)	49.0	(47.6–50.0)			0.174	0.090^††^	49.0	(47.6–50.5)	0.262^||^	49.4	(48.5–50.5)	0.243^||^	49.2	(47.9–51.0)	0.103^||^	47.6	(46.3–49.5)	0.153^||^
One month checkup weight (g)	4233	(3833–4534)			0.058	0.573^††^	4210	(3830–4675)	0.793^||^	4185	(3965–4320)	0.738^||^	4260	(3774–4684)	0.495^||^	3840	(3694–4165)	**0.028**^||^
One month checkup height (cm)	53.0	(52.0–54.7)			0.063	0.539^††^	53.0	(52.0–55.0)	0.576^||^	53.3	(52.0–54.5)	0.647^||^	53.0	(51.9–55.0)	0.921^||^	52.3	(51.5–53.6)	0.242^||^
Family history of atopic dermatitis						0.270^||^			0.718^¶^			1.000^¶^			0.474^¶^			0.874^‡‡^
Negative	66	(68.8)	8.0	(4.0–11.3)			37	(56.1)		11	(16.7)		30	(45.5)		5	(7.6)	
Positive	30	(31.3)	9.0	(4.5–14.0)			18	(60.0)		5	(16.7)		16	(53.3)		2	(6.7)	
Infants’ birth season						**0.039**^§§^			0.247^¶^			0.670^¶^			0.099^¶^			0.475^¶^
Spring (March–May)	48	(50.0)	7.5	(2.0–11.8)			24	(50.0)		8	(16.7)		18	(37.5)		2	(4.2)	
Summer (June–August)	36	(37.5)	8.0	(6.0–11.8)			22	(61.1)		7	(19.4)		20	(55.6)		4	(11.1)	
Autumn (September–November)	12	(12.5)	11.0	(7.5–13.8)			9	(75.0)		1	(8.3)		8	(66.7)		1	(8.3)	
Skin barrier function of forehead																		
SCH^||||^	62.0	(51.1–70.6)			− 0.107	0.299^††^	62.3	(48.7–71.7)	0.859^||^	61.5	(39.7–68.5)	0.205^||^	67.5	(58.2–73.5)	0.056^||^	51.0	(35.3–69.0)	0.147^||^
Sebum	86.3	(55.1–122.3)			− 0.058	0.578^††^	86.5	(58.0–117.0)	0.920^||^	82.0	(64.5–115.6)	0.727^||^	91.3	(61.9–118.8)	0.585^||^	58.0	(46.5–100.0)	0.207^||^
Skin pH	4.9	(4.6–5.3)			− 0.004	0.969^††^	4.8	(4.6–5.3)	0.441^||^	4.9	(4.5–5.3)	0.749^||^	4.8	(4.6–5.3)	0.454^||^	4.9	(4.5–5.2)	0.833^||^
TEWL^¶¶^(g/m^2^ per hour)	10.5	(8.2–13.8)			0.244	**0.017**^††^	11.1	(8.8–14.6)	0.075^||^	11.6	(9.2–15.9)	0.131^||^	11.6	(9.1–15.3)	**0.033**^||^	7.8	(7.1–10.1)	**0.036**^||^
Albumin density^†††^ (ng/cm^2^)	2076	(1803–2387)			0.265	**0.009**^††^	2159	(1859–2545)	**0.032**^||^	2083	(1687–2401)	0.735^||^	2181	(1961–2664)	**0.001**^||^	2115	(1717–3097)	0.647^||^

Significant values are in bold.

^†^Infant facial skin visual assessment tool (score 0–53), ^‡^≥ 8 points in case of infant facial skin visual assessment tool, ^§^ nterquartile range (25% tile–75% tile), ^||^Mann–Whitney U test, ^¶^Chi-square test, ^††^Spearman's rank correlation coefficient, ^‡‡^Fisher's exact test,^§§^Kruskal–Wallis test, ^||||^Stratum corneum hydration, ^¶¶^Transepidermal water loss, ^†††^Evaluating by skin blotting methods.

*IFSAT* infant facial skin visual assessment tool, *IQR* interquartile range, *SCH* stratum corneum hydration, *TEWL* transepidermal water loss.

As shown in Table 1, infants born by cesarean section were significantly more likely to have forehead dryness (*p* = 0.007). Infants with forehead dryness had significantly lower birth weights and 1-month checkup weights (*p* = 0.049 and 0.028, respectively). Infants born in the spring (March–May) had significantly lower scores, whereas those born in the autumn (September–November) had significantly higher scores (*p* = 0.039).

The temperature and humidity [mean ± standard deviation (SD)] at the time of skin barrier function measurement were 25.9 ± 2.0 °C and 59.6 ± 9.1%, respectively. Regarding skin barrier function, TEWL was significantly associated with infantile facial skin problems. TEWL was positively correlated with IFSAT scores (*p* = 0.017). Moreover, high TEWL was significantly associated with papules on the forehead (*p* = 0.033), whereas low TEWL was significantly associated with forehead dryness (*p* = 0.036).

The albumin density measured using the skin blotting method was positively correlated with IFSAT scores (*p* = 0.009). High albumin density was significantly associated with severe facial skin problems and forehead papules (*p* = 0.032 and 0.001, respectively).

### Four inflammatory cytokine signals by skin blotting method

Table 2 summarizes the association between detecting four inflammatory cytokine signals by skin blotting and infantile facial skin problems. Positive signals for IL-4, IL-6, IL-8, and IL-17 were found in 40 (41.7%), 25 (26.0%), 46 (47.9%), and 68 (70.8%) infants, respectively. A positive IL-8 signal was significantly associated with high IFSAT scores, severe facial skin problems, and forehead papules (*p* = 0.006, 0.006, and 0.043, respectively).

**Table 2:** Relationship of four inflammatory cytokine signals by skin blotting method with IFSAT scores, severe facial skin problems, forehead erythema, forehead papules, and forehead dryness (n = 96).

	Total (n = 96)		IFSAT^†^ scores (n = 96)		Severe facial skin problems^‡^ (n = 55, 57.3%)			Forehead erythema (n = 16, 16.7%)			Forehead papules (n = 46, 47.9%)			Forehead dryness (n = 7, 7.3%)		
	n	(%)	*ρ*	*p*-value	n	(%)	*p*-value	n	(%)	*p*-value	n	(%)	*p*-value	n	(%)	*p*-value
IL-4 signal			− 0.164	0.111^||^			0.222^¶^			0.355^¶^			0.629^¶^			0.465^††^
Positive	40	(41.7)			20	(50.0)		5	(12.5)		18	(45.0)		2	(5.0)	
Negative	56	(58.3)			35	(62.5)		11	(19.6)		28	(50.0)		5	(8.9)	
IL-6 signal			0.046	0.660^||^			0.879^¶^			0.467^††^			0.635^¶^			0.292^††^
Positive	25	(26.0)			14	(56.0)		3	(12.0)		13	(52.0)		3	(12.0)	
Negative	71	(74.0)			41	(57.7)		13	(18.3)		33	(46.5)		4	(5.6)	
IL-8 signal			0.279	**0.006**^||^			**0.006**^¶^			0.715^¶^			**0.043**^¶^			0.612^††^
Positive	46	(47.9)			33	(71.7)		7	(15.2)		27	(58.7)		4	(8.7)	
Negative	50	(52.1)			22	(44.0)		9	(18.0)		19	(38.0)		3	(6.0)	
IL-17 signal			− 0.049	0.639^||^			0.664^¶^			0.422^¶^			0.524^¶^			0.971^††^
Positive	68	(70.8)			38	(55.9)		10	(14.7)		34	(50.0)		5	(7.4)	
Negative	28	(29.2)			17	(60.7)		6	(21.4)		12	(42.9)		2	(7.1)	

Significant values are in bold.

^†^Infant facial skin visual assessment tool (score 0–53), ^‡^≥ 8 points in case of infant facial skin visual assessment tool, ^§^Interquartile range (25% tile–75% tile), ^||^Spearman’s rank correlation coefficient, ^¶^Chi-square test, ^††^Fisher’s exact test.

*IFSAT* infant facial skin visual assessment tool, *IL* interleukin.

### Relationship of four inflammatory cytokine signals with IFSAT scores by multiple regression analysis

Table 3 summarizes the relationship of four inflammatory cytokine signals with IFSAT scores by multiple regression analysis. In the multiple regression analysis, the following independent variables were selected: 1) four inflammatory cytokine signals (IL-4, IL-6, IL-8, and IL-17); 2) birth season, which was significantly associated with the IFSAT scores; and 3) family history of atopic dermatitis, which was not significantly associated with the IFSAT scores but was speculated to influence skin problems in infants. Multiple regression analysis showed no significant association between IL-4, IL-6, IL-8, and IL-17 signals and IFSAT scores (*p* = 0.485, 0.278, 0.085, and 0.103, respectively).

**Table 3:** Relationship of four inflammatory cytokine signals with IFSAT scores by multiple regression analysis (n = 96).

	IFSAT^†^ scores	
	*β*^‡^	*p*-value
Four inflammatory cytokines signals^§^		
IL-4 signal^||^	− 0.07	0.485
IL-6 signal^||^	0.12	0.278
IL-8 signal^||^	0.19	0.085
IL-17 signal^||^	− 0.17	0.103
Participants’ characteristics		
Family history of atopic dermatitis^||^	0.09	0.353
Birth seasons		
Spring (March–May)	*Reference*	
Summer (June–August)	0.09	0.420
Autumn (September–November)	0.18	0.136
*R*^2^		0.13
Adjusted *R*^2^		0.06
Durbin–Watson ratio		1.88

^‡^Standardized coefficient beta, ^§^ Evaluating by skin blotting methods, ^||^Negative = 0, Positive = 1.

*IFSAT* infant facial skin visual assessment tool, *IL* interleukin.

#### *Malassezia*

In this study, the *Malassezia* genus was detected in all 96 infants in the form of the following 16 *Malassezia* species: *M. globosa*, *M. restricta*, *M. sympodialis*, *M. arunalokei*, *M. obtusa*, *M. furfur*, *M. slooffiae*, *M. pachydermatis*, *M. caprae*, *M. yamatoensis*, *M. dermatis*, *M. japonica*, *M. equina*, *M. nana*, *M. vespertilionis*, and *M. cuniculi*.

Of these 16 *Malassezia* species, six species (*M. globosa*, *M. restricta*, *M. sympodialis*, *M. arunalokei*, *M. obtusa*, and *M. furfur)* accounted for a median percentage > 0.001% of the total fungal population and were analyzed further.

Table 4 presents the association between the presence of *Malassezia* and infantile facial skin problems. The percentage of *M. arunalokei* was significantly lower in the dry forehead group (*p* = 0.006).

**Table 4:** Relationship of *Malassezia* with IFSAT scores, severe facial skin problems, forehead erythema, forehead papules, and forehead dryness (n = 96).

	Total (n = 96)		IFSAT^†^ scores (n = 96)		Severe facial skin problems^‡^ (n = 55, 57.3%)			Forehead erythema (n = 16, 16.7%)			Forehead papules (n = 46, 47.9%)			Forehead dryness (n = 7, 7.3%)		
	Median (IQR^§^)		*ρ*	*p*-value^*||*^	median (IQR^§^)		*p*-value^*||*¶^	median (IQR^§^)		*p*-value^*||*¶^	median (IQR^§^)		*p*-value^*||*¶^	median (IQR^§^)		*p*-value^*||*¶^
*Malassezia* genus (%)^††^																
Total* Malassezia*	37.27	(4.49–61.17)	0.04	0.731	40.25	(6.29–64.12)	0.584	41.55	(8.13–77.97)	0.323	46.96	(9.71–64.85)	0.233	2.79	(0.73–34.61)	0.072
*Malassezia* species (%)^††^																
* M. globosa*	8.15	(0.79–22.24)	0.10	0.323	9.01	(2.04–24.50)	0.314	16.64	(2.89–30.33)	0.205	9.43	(1.87–21.97)	0.208	2.04	(0.14–6.43)	0.089
* M. restricta*	3.37	(0.63–15.91)	0.06	0.591	4.69	(0.85–17.14)	0.223	3.35	(1.14–12.20)	0.856	5.37	(1.04–19.72)	0.141	0.52	(0.16–6.47)	0.125
* M. sympodialis*	0.46	(0.03–3.45)	− 0.07	0.468	0.58	(0.01–3.11)	0.619	0.70	(0.04–7.50)	0.619	0.35	(0.02–3.72)	0.572	0.23	(0.03–0.80)	0.525
* M. arunalokei*	0.52	(0.06–2.11)	0.01	0.930	0.61	(0.10–2.19)	0.388	0.53	(0.12–3.86)	0.558	0.97	(0.21–2.60)	0.056	0.02	(0.01–0.14)	**0.006**
* M. obtusa*	0.05	(0.00–0.39)	0.07	0.518	0.06	(0.00–0.42)	0.493	0.06	(0.00–0.27)	0.823	0.05	(0.01–0.35)	0.702	0.02	(0.00–19.15)	0.781
* M. furfur*	0.02	(0.00–0.27)	0.07	0.522	0.04	(0.00–0.31)	0.231	0.02	(0.00–0.35)	0.939	0.06	(0.00–0.33)	0.119	0.00	(0.00–0.18)	0.336

Significant values are in bold.

^†^Infant facial skin visual assessment tool (score 0–53), ^‡^≥ 8 points in case of infant facial skin visual assessment tool, ^§^ Interquartile range (25% tile–75% tile), ^||^Spearman’s rank correlation coefficient, ^¶^Mann–Whitney U test, ^††^Percentage of total fungal populations was calculated.

*IFSAT* infant facial skin visual assessment tool, *IQR* interquartile range.

Although not shown in Table 4, no significant association was found between a family history of atopic dermatitis and the *Malassezia* genus, *M. globosa*, *M. restricta*, *M. sympodialis*, *M. arunalokei*, *M. obtusa*, and *M. furfur* (*p* = 0.372, 0.769, 0.784, 0.217, 0.414, 0.272, and 0.603, respectively).

Supplementary Table 1 shows the percentage incidence of 16 *Malassezia* species detected in this study with reference to the total fungal population.

### Relationship of *Malassezia* with four inflammatory cytokine signals by multiple logistic regression analysis

Table 5 presents the relationship of *Malassezia* with four inflammatory cytokine signals (IL-4, IL-6, IL-8, and IL-17) found in multiple logistic regression analysis. Because the *Malassezia* genus and each of its species was evaluated relative to the total fungal population, they were analyzed in separate models. Additionally, a family history of atopic dermatitis and birth season were selected as independent variables. In the analysis, before adjustment for a family history of atopic dermatitis and birth season, a significant association between *M. sympodialis* and IL-8 signals was found (*p* = 0.037). However, logistic regression analysis adjusted for background factors showed no significant association between *Malassezia* and all inflammatory cytokine signals.

**Table 5:** Relationship of *Malassezia* with four inflammatory cytokine signals by multiple logistic regression analysis (n = 96).

	IL-4 signal					IL-6 signal					IL-8 signal					IL-17 signal				
	OR	(95% CI^†^)	AOR^‡^	(95% CI^†^)	*p*-value	OR	(95% CI^†^)	AOR^‡^	(95% CI^†^)	*p*-value	OR	(95% CI^†^)	AOR^‡^	(95% CI^†^)	*p*-value	OR	(95% CI^†^)	AOR^‡^	(95% CI^†^)	*p*-value
*Malassezia* genus (%)^§^																				
Total* Malassezia*	0.99	(0.98–1.01)	0.99	(0.98–1.01)	0.351	0.99	(0.97–1.01)	0.99	(0.97–1.01)	0.181	1.00	(0.99–1.01)	1.00	(0.99–1.02)	0.904	1.01	(0.99–1.03)	1.01	(1.00–1.03)	0.168
*Malassezia* species (%)^§^																				
* M. globosa*	1.01	(0.98–1.04)	1.00	(0.97–1.03)	0.959	0.98	(0.95–1.02)	0.98	(0.94–1.02)	0.263	0.98	(0.95–1.01)	0.99	(0.96–1.02)	0.528	1.01	(0.98–1.04)	1.02	(0.98–1.05)	0.378
* M. restricta*	0.98	(0.95–1.01)	0.99	(0.95–1.02)	0.381	0.99	(0.96–1.02)	0.99	(0.95–1.02)	0.440	1.02	(0.99–1.06)	1.02	(0.99–1.05)	0.214	1.00	(0.97–1.03)	1.00	(0.97–1.03)	0.957
* M. sympodialis*	1.01	(0.98–1.05)	1.01	(0.97–1.05)	0.575	0.83	(0.68–1.03)	0.83	(0.67–1.02)	0.081	0.94	(0.89–1.00)*	0.94	(0.88–1.00)	0.059	1.02	(0.97–1.06)	1.02	(0.98–1.07)	0.349
* M. arunalokei*	0.89	(0.77–1.02)	0.89	(0.77–1.03)	0.112	1.05	(0.98–1.12)	1.06	(0.99–1.14)	0.106	1.03	(0.96–1.09)	1.01	(0.94–1.09)	0.688	1.04	(0.95–1.13)	1.04	(0.94–1.14)	0.448
* M. obtusa*	0.98	(0.93–1.04)	0.98	(0.93–1.04)	0.458	1.00	(0.95–1.06)	0.99	(0.94–1.05)	0.853	1.05	(0.98–1.12)	1.06	(0.99–1.13)	0.105	1.06	(0.96–1.17)	1.06	(0.96–1.18)	0.243
* M. furfur*	0.99	(0.95–1.04)	1.00	(0.95–1.04)	0.848	1.01	(0.97–1.05)	1.01	(0.97–1.06)	0.544	1.01	(0.97–1.05)	1.00	(0.96–1.05)	0.882	1.00	(0.96–1.05)	1.00	(0.95–1.05)	0.945

^†^*CI* confidence interval, ^‡^*AOR* Adjusted Odds Ratio, Multiple logistic regression analysis was adjusted for the variables of family history of atopic dermatitis and birth season, ^§^Percentage of total fungal populations was calculated, *p < 0.05.

The relationship between the four inflammatory cytokines and *Malassezia* presence in six infants with IFSAT scores of 0 is shown in Supplementary Table 2.

## Discussion

There are three novel aspects to our findings in this study. First, an association between infantile facial skin problems and inflammatory cytokines was revealed. Second, genetic analysis was used to accurately investigate the relationship between facial skin problems and the presence of various *Malassezia* species in infants. Third, the association between *Malassezia* and inflammatory cytokines in infants was analyzed. Our data show a correlation between inflammatory cytokines and *Malassezia* in infantile facial skin problems and will help elucidate the mechanism of the onset of facial skin problems in infants.

This study has three main findings. First, there was no significant association between IFSAT scores and inflammatory cytokine signals using the skin blotting method. Nevertheless, infants with positive IL-8 signals were more likely to have severe facial skin problems and forehead papules. Second, no significant association was observed between IFSAT scores and *Malassezia*; however, infants with forehead dryness showed a lower percentage of *M. arunalokei* in the total fungal population. Finally, contrary to the hypothesis, no significant association was found between inflammatory cytokines and *Malassezia*.

The participants in this study may be relatively representative of the general infant population in Japan. The percentage of infants defined as having severe facial skin problems, and the median IFSAT score in this study were almost the same as in a previous study^32^. Infants with a family history of atopic dermatitis accounted for 31.3% of patients in this study, almost the same as that reported by a previous study conducted in Japan (27.1%)^6^. Additionally, TEWL, stratum corneum hydration (SCH), sebum secretion, and skin pH measurements were similar to those of previous studies^33,34^. This study recruited low-risk infants, who are generally healthy. Therefore, the participants in this study might have had similar characteristics to those in previous studies conducted in Japan.

This study showed that infants with positive IL-8 signals, measured using the skin blotting method, were more likely to have severe facial skin problems and forehead papules. The reason why only IL-8 was associated with infantile facial skin problems among the four inflammatory cytokines evaluated in this study may be based on whether it is produced by natural or acquired immunity. There are two types of immunity in the skin: natural and acquired. Generally, natural immunity responds more rapidly than acquired immunity. In the skin, IL-8 is mainly produced by keratinocytes and is responsible for natural immunity; IL-4 and IL-17 are produced by cells responsible for acquired immunity, whereas IL-6 is produced by cells responsible for both natural and acquired immunity^35^. Of the four inflammatory cytokines evaluated in this study, IL-8 was produced most rapidly, suggesting that the presence of IL-8 signals is associated with infantile facial skin problems at the time of the study. Therefore, IL-8 may be a suitable index to evaluate the degree of real-time inflammation in infantile facial skin problems.

The papules are thought to be caused by increased capillary permeability, which allows plasma to leak out of blood vessels, causing the skin to swell^36^. Of the four inflammatory cytokines investigated in this study (IL-4, IL-6, IL-8, and IL-17), only IL-8 induced neutrophils, which normally reside in blood vessels, to migrate to the site of inflammation^35^. Since IL-8 is thought to increase the permeability of blood vessels so that leukocytes can pass through the vessel walls, there may have been a significant association between forehead papules and IL-8. Although this is a cross-sectional study and the causal relationship is unclear, the results suggest that IL-8 may be involved in the development of forehead papules in infants.

This study showed no significant association between IFSAT scores and *Malassezia*, but infants with forehead dryness had a lower percentage of *M. arunalokei* in the total fungal population. The absence of a statistically significant association between IFSAT scores and *Malassezia* presence in this study may be because the IFSAT score is a tool to assess the severity of skin conditions and does not assess the specific nature of the skin disease. *Malassezia* has been suggested to be a cause or aggravating factor in various skin problems, such as infantile seborrheic eczema, neonatal acne, and atopic dermatitis^8–16^. However, the *Malassezia* species associated with each skin disease are unclear from the literature. Therefore, the *Malassezia* species that triggers each skin disease may vary, rather than a single specific *Malassezia* species being involved in all infantile facial skin problems. Therefore, the results of this study suggest that a separate analysis of each skin disease, rather than an analysis by severity, is necessary when examining the association between infantile facial skin problems and *Malassezia*. In the future, it will be necessary to expand the sample size of infants without skin problems. This would help in further elucidating the differences in *Malassezia* in each skin disease and the composition of *Malassezia* species in areas that are not typically affected by *Malassezia*.

*M. arunalokei* is a new *Malassezia* species discovered in 2016 in a mild-to-moderate seborrheic dermatitis case of a teenage male patient^19^. It is still unclear how *M. arunalokei* differs from other *Malassezia* species. Although the number of infants with dry foreheads in this study was small, the fact that *M. arunalokei* was detected in infants as young as 1-month-old and was associated with dry foreheads is a novel finding. The results of this study suggest that the absence or low percentage of *M. arunalokei* may be associated with skin dryness in infants.

Contrary to the proposed hypothesis, this study found no significant association between inflammatory cytokines and *Malassezia*. The results of this study differ from those of previous studies that reported such an association^23–27^. Previous studies used T-cells or normal human epidermal keratinocytes, whereas this is the first study to investigate the relationship between inflammatory cytokines and *Malassezia* in infantile skin. In human skin, various cells produce inflammatory cytokines, including keratinocytes, T cells, and macrophages. The fact that *Malassezia* has been detected in various skin diseases with different pathogenic mechanisms, such as infantile seborrheic eczema, neonatal acne, and atopic dermatitis, in previous studies suggests that *Malassezia* may be recognized by many skin immune cells and activate pathogen elimination mechanisms, thus triggering inflammation^8–16^. Therefore, the absence of an association between *Malassezia* and inflammatory cytokines in this study may be because inflammatory cytokines were produced from multiple skin immune cells inside the infant’s skin for *Malassezia* infection, which did not lead to the production of specific types of inflammatory cytokines. The results of this study suggest that the association between *Malassezia* and inflammatory cytokines may differ between experiments using skin cells and investigations using actual human skin. Therefore, further studies are needed to reveal the association between *Malassezia* and inflammatory cytokines in human skin.

The present study has four main limitations. First, a scale was used to assess skin problems in this study, and no medical diagnosis was made. However, the severity of facial skin problems was assessed in infants, and this may be a strength of this study. Second, this study permitted daily skincare for infants on the survey day. Therefore, such skincare might have influenced the skin blotting results. To maintain the cleanliness of the infant’s skin, no skincare restrictions were implemented on the day of the study. Third, skin blotting could not ascertain which immune cells produced the cytokines. Therefore, caution is necessary when considering infantile skin immunity or mechanisms of infantile skin problems based on the results of this study. Finally, this study only evaluated the fungal population on the skin. Generally, skin problems are caused by an imbalance of bacteria and fungi on the skin. Therefore, clarifying the relationship between infantile facial skin problems by considering both bacteria and fungi is warranted in the future.

Despite these limitations, a strength of this study was that it investigated the association between inflammatory cytokines and *Malassezia* in infants with facial skin problems with a relatively large sample size. Therefore, the results of this study may be generalizable to all infants aged approximately 1 month in Japan. However, even within Japan, climatic differences may have some effect on inflammatory cytokines and *Malassezia* in the skin; thus, the findings of this study should still be interpreted with caution.

The results showed that the presence of IL-8 signals was associated with infantile facial skin problems. Although it is difficult to apply the study results to clinical practice directly, the findings highlight the need to focus on the association between IL-8 and preventing infantile facial skin problems. Therefore, elucidating the causal relationship between IL-8 and infantile facial skin problems in a longitudinal study is necessary.

In conclusion, to the best of our knowledge, this is the first study to investigate how inflammatory cytokines, assessed by the skin blotting method, and *Malassezia* are associated with infantile facial skin problems. The results of this study suggest that IL-8 is a suitable index for evaluating the degree of real-time inflammation in infantile facial skin problems. However, this study did not find an association between *Malassezia* and infantile facial skin problems or between inflammatory cytokines and *Malassezia*. As this was a cross-sectional study, the causal relationship between IL-8 and infantile facial skin problems was not determined. Thus, a longitudinal study may be necessary to clarify this causal relationship. Further research is warranted to investigate the relationship between *Malassezia* and infantile skin disease and the infant skin’s immune response to *Malassezia* infection.

## Methods

### Study design and participants

This cross-sectional study was conducted from April to November of 2021 in Tokyo and surrounding areas in Japan. Healthy infants aged approximately 1 month and their parents were recruited as follows: (1) a researcher approached parents who visited the pediatric department of a general hospital for their infants’ 1-month medical checkup; (2) a snowball sampling method was employed; (3) posters/leaflets were displayed in the pediatric and obstetric outpatient departments of a general hospital, an obstetrics clinic, and a pediatric clinic; and (4) the study was announced on the laboratory website. The inclusion criteria were as follows: (1) infants whose parents could speak Japanese, and (2) infants with parents aged ≥ 20 years. Infants with congenital skin diseases were excluded. Data were collected by a single researcher after informed consent was obtained from the parents.

### Procedures

The researcher explained the study details to parents at their infant’s 1-month medical checkup or via e-mail to pregnant/postpartum women interested in participating. Data were collected at a location of the participant’s choice (the hospital examination room, the participant’s house, or a laboratory room at the University of Tokyo) when the infant was 1 month of age. First, the infant’s skin condition was examined using an IFSAT; second, the skin barrier function was determined; third, samples of skin inflammatory cytokines were collected using the skin blotting method; and finally, *Malassezia* samples were obtained by swabbing.

The predominant site for collecting infantile seborrheic dermatitis samples is from the eyebrows to the scalp^37^; therefore, skin barrier function measurements, skin blotting, and *Malassezia* sample collection were conducted on the forehead, avoiding the hair area.

Parents completed an online questionnaire on their infants’ attributes while skin barrier function was examined.

### Variables

#### Skin problems

Infantile facial skin problems were evaluated using the IFSAT, a scale developed in Japan that rates skin conditions between 0 and 53 points, with higher scores indicating more severe skin problems and longer healing periods. The reliability and validity of the IFSAT score in infants aged 1 month were confirmed during its development. In the IFSAT developmental study^32^, a score of 8 points was set as the cut-off and criterion necessary for diagnosis and treatment. Thus, in this study, a score of 8 points or higher indicated severe facial skin problems. The tool includes four items about skin problems (erythema, papules, dryness, and exudate/yellow scaling) and nine items about particular facial areas (the scalp and hairline, right forehead, left forehead, eyebrows/between the eyebrows/eyes, nose, right cheek, left cheek, around the mouth and jaw, and ears). This tool’s scoring system combined two facial areas (right forehead and left forehead) into one area (forehead), and the scores for erythema forehead, papules forehead, papules left cheek, papules right cheek, papules mouth, dryness, and exudate/yellow scaling were doubled.

#### Inflammatory cytokines and albumin levels

Skin blotting was performed similar to that reported in a previous study^30^. First, a 1 × 1 cm nitrocellulose membrane (Bio-Rad Laboratories, Hercules, CA, USA) was hydrated with saline and attached to the infantile forehead for 10 min. Next, immunostaining of inflammatory cytokines and albumin on membranes was performed using SNAP i.d. 2.0 (Merck KGaA, Darmstadt, Germany). Nitrocellulose membranes were divided into three pieces: one for double staining for albumin and IL-17; one for double staining for IL-8 and IL-4, and one for single staining for IL-6.

As a positive control, the nitrocellulose membrane was blotted with a mixture of recombinant proteins: recombinant albumin (A9731-1G; Sigma-Aldrich, St. Louis, MO, USA) and recombinant IL-17 (NBP2-35040; Novus Biologicals, CO, USA); recombinant IL-8 (NBC1-18459; Novus Biologicals) and recombinant IL-4 (NBP2-52393; Novus Biologicals); and a single recombinant protein IL-6 (NBP2-52256; Novus Biologicals). As a negative control, a nitrocellulose membrane was prepared without blotting.

In the staining process, the membranes were first blocked for 10 min with Blocking One (Nacalai Tesque Inc., Kyoto, Japan). Then, staining was performed for each biomarker. Albumin was detected following the reaction for 10 min with a primary antibody bound to an alkaline phosphatase (ALP) label (dilution 1:500; A80-229AP; Bethyl Laboratories Inc., Montgomery, TX, USA). IL-17, IL-8, IL-4, and IL-6 levels were detected following the reaction for 10 min with primary antibodies, i.e., anti-IL-17 antibody (dilution 1:2000; PRS4887; Merck KGaA), anti-IL-8 antibody (dilution 1:4000; WH0003576M5; Merck KGaA), anti-IL-4 antibody (dilution 1:2000; 12227S; Cell Signaling Technology, Danvers, MA, USA), or anti-IL-6 antibody (dilution 1:1000; 12153S; Cell Signaling Technology). This was followed by a reaction with a secondary antibody labeled with ALP (dilution 1:1000; Jackson ImmunoResearch, West Grove, PA, USA) or horseradish peroxidase (dilution 1:1000; Jackson ImmunoResearch). Immunoreactivity was visualized using ALP (BioFX Chemiluminescent AP Microwell/Membrane Substrate, Ultra Sensitive; SurModics, Eden Prairie, MN, USA) and peroxidase (Luminata Forte; Merck KGaA) chemiluminescent substrates and recorded using a chemiluminescence imaging device (LumiCube Liponics, Tokyo, Japan).

The presence of IL-4, IL-6, IL-8, and IL-17 signals was evaluated by banalization using threshold intensity ([mean + 2 × SD]) of the signal intensity of the negative control sample, as calculated by an image analysis software (Image J version 1.51 s; National Institutes of Health, Bethesda, MD, USA). Albumin level was calculated as follows: (1) the area and average brightness of each nitrocellulose membrane were measured within 1 mm of the edge; and (2) the albumin quantity per cm^2^ of each nitrocellulose membrane was calculated using ImageJ (version 1.51 s; National Institutes of Health) and the following formula:

$$\frac{\text{AB \, of \, target - AB \, of \, NC}}{\text{TB \, of \, PC}}\times QP \times Constant$$

where *AB* is the average brightness; *TB*, total brightness = AB × area; *NC*, negative control; *PC*, positive control; *QP*, quantity of positive control = 200 ng albumin; *Constant*, 174,232.13 pixel/cm^2^.

#### *Malassezia* species identification

*Malassezia* samples were obtained from the forehead by swabbing a 2 × 4 cm skin area for 30 s, according to a previous study^38^. DNA was extracted from these swab samples using the QIAamp DNA Mini Kit (Qiagen, Hilden, Germany), according to the manufacturer’s instructions. After DNA extraction, the fungal internal transcribed spacer (ITS) sequence was amplified by amplicon polymerase chain reaction (PCR). The primers for ITS1 and ITS2 were ITS1, 5′-GGAAGTAAAAGTCGTAACAAGG-3′ and 5′-GCTGCGTTCTTCATCGATGC-3′; ITS2, 5′-GCATCGATGAAGAACGCAGC-3′ and 5′-TCCTCCGCTTATTGATATGC-3′ (forward and reverse, respectively). These are common ITS sequencing primers for fungi^39^ and were used in a previous study that analyzed fungi on the skin^40^. The amplified fragments were then sequenced using the ION PGM sequencer system (Thermo Fisher Scientific, Waltham, MA, USA). The determined ITS1 and ITS2 sequences were subjected to homology searches for a BLAST analysis against the NCBI database (National Center for Biotechnology Information, Bethesda, MD, USA), and the highest homology fungal species were assigned. DNA extraction, PCR amplification, next-generation sequencing, and bioinformatics analysis were performed by World Fusion Co. Ltd. (Tokyo, Japan).

For analysis, the percentage of *Malassezia* genus and each *Malassezia* species in the total fungal population of each sample and the Simpson’s index were calculated. The Simpson’s index represents species diversity and can be between zero and one, with a value closer to one indicating greater diversity.

#### Demographic data of infants

One parent of each infant completed an online questionnaire regarding the following: gender of the infant, birth weight, birth height, gestational age (weeks), mode of delivery, mother’s parity, 1-month checkup weight and height, parents’ family history of atopic dermatitis (within the first degree of consanguinity), and infant’s birth season.

#### Skin barrier function

Skin barrier function was examined using TEWL (VapoMeter SWL-5001; Delfin Technologies, Kuopio, Finland), SCH (Mobile moisture HP10; Courage + Khazaka, Electronic GmbH, Köln, Germany), sebum secretion (SM815; Courage + Khazaka), and skin pH (Skin-pH-Meter PH905; Courage + Khazaka). Skin barrier function measurements were performed by a single, well-trained researcher with experience in measuring infants’ skin barrier function.

A previous study has shown that the intensity of albumin detected by skin blotting correlates with TEWL^31^. Hence, the albumin results of skin blotting were evaluated as a skin barrier function in this study.

### Statistical analysis

To evaluate the association between inflammatory cytokine signals and infantile facial skin problems, Spearman’s rank correlation coefficient was used to analyze the associations with IFSAT scores, and the chi-square test and Fisher’s exact test were used to analyze the associations with severe facial skin problems, forehead erythema, forehead papules, and forehead dryness.

To evaluate the relationship between the percentage of *Malassezia* in the total fungal population and infantile facial skin problems, Spearman’s rank correlation coefficient was used to analyze the association between IFSAT scores. The Mann–Whitney *U* test was performed to analyze the association between severe facial skin problems, forehead erythema, forehead papules, and forehead dryness.

For the relationship of inflammatory cytokines with IFSAT scores, a multiple regression analysis adjusted for background factors (factors at birth, at 1-month checkup, and family) was performed. For the relationship of *Malassezia* with inflammatory cytokines, a logistic regression analysis adjusted for the same background factors was performed. Background factors for multivariate analysis were selected with a *p*-value of < 0.05 in the bivariate analysis or important factors that may affect infantile facial skin problems. Multicollinearity was evaluated using Spearman’s rank correlation coefficient (*ρ*). When correlations (i.e., |*ρ*| ≥ 0.6) were detected, only one of these variables was included in the multivariate analysis.

Two-tailed *p*-values of < 0.05 were considered to indicate statistical significance. All analyses were performed using SPSS version 28.0 for Windows (IBM Corp., Armonk, NY, USA).

### Ethical considerations

A signed written informed consent form was obtained from the infants’ parents before study participation. The study was performed in accordance with the principles embodied in the World Medical Association’s Declaration of Helsinki and was approved by the Ethics Committee of the Graduate School of Medicine at the University of Tokyo (Approval Number: 2020349NI).

## Supplementary Information

Supplementary Tables.

## Data Availability

The datasets generated during and analyzed during the current study are available from the corresponding author upon reasonable request.
