# Supplementary material for: Relationship between facial skin problems with a focus on inflammatory cytokines and the presence of Malassezia in 1-month-old infants
Source: Sci Rep. 2023 Mar 28;13:5041. doi: 10.1038/s41598-023-31949-2 (PMC10049982; doi:10.1038/s41598-023-31949-2)
Supplement: Supplementary file 1 — Supplementary Tables. [file 41598_2023_31949_MOESM1_ESM.docx]

**Relationship between facial skin problems with a focus on inflammatory cytokines and the presence of *Malassezia* in 1-month-old infants**

Satsuki Shimizu^1^, Kaori Yonezawa^1,2*^, Megumi Haruna^1,2^, Emi Sasagawa^1,2^, Yuriko Usui^1,2^, Takeo Minematsu^2,3,4^, Sachi Higuchi^5^

^1^Department of Midwifery and Women’s Health, Division of Health Sciences and Nursing, Graduate School of Medicine, The University of Tokyo, 7-3-1 Hongo, Bunkyo-ku, Tokyo 113-0033, Japan.

^2^Global Nursing Research Center, Graduate School of Medicine, The University of Tokyo, 7-3-1 Hongo, Bunkyo-ku, Tokyo 113-0033, Japan.

^3^Department of Skincare Science, Graduate School of Medicine, The University of Tokyo, 7-3-1 Hongo, Bunkyo-ku, Tokyo 113-0033, Japan.

^4^Ishikawa Prefectural Nursing University, 1-1 Gakuendai, Kahoku-city, Ishikawa 929-1210, Japan.

^5^Department of Midwifery, Oita University of Nursing and Health Sciences, 2944-9 Megusuno, Oita-City, Oita 870-1201, Japan.

***Corresponding author:**

Kaori Yonezawa,

Department of Midwifery and Women’s Health,

Division of Health Sciences and Nursing, Graduate School of Medicine,

The University of Tokyo, 7-3-1 Hongo, Bunkyo-ku, Tokyo 113-0033, Japan.

Tel: +81-3-5841-3396

Email: [kaoriyone@m.u-tokyo.ac.jp](mailto:kaoriyone@m.u-tokyo.ac.jp)

| **Supplementary Table 1. Percentage of *Malassezia* in total fungal population** | | | | | |
| --- | --- | --- | --- | --- | --- |
|  | Total (n=96) | | | | |
|  | median (IQR^†^) | | | | |
| ***Malassezia* genus** (%)^††^ |  |  |  |  |  |
| Total *Malassezia* | 37.27 | (4.49 | – | 61.17) |  |
| ***Malassezia* species** (%)^‡^ |  |  |  |  |  |
| *M. globosa* | 8.15 | (0.79 | – | 22.24) |  |
| *M. restricta* | 3.37 | (0.63 | – | 15.91) |  |
| *M. sympodialis* | 0.46 | (0.03 | – | 3.45) |  |
| *M. arunalokei* | 0.52 | (0.06 | – | 2.11) |  |
| *M. obtusa* | 0.05 | (0.00 | – | 0.39) |  |
| *M. furfur* | 0.02 | (0.00 | – | 0.27) |  |
| *M. slooffiae* | 0.00 | (0.00 | – | 0.00) |  |
| *M. pachydermatis* | 0.00 | (0.00 | – | 0.02) |  |
| *M. caprae* | 0.00 | (0.00 | – | 0.02) |  |
| *M. yamatoensis* | 0.00 | (0.00 | – | 0.01) |  |
| *M. dermatis* | 0.00 | (0.00 | – | 0.01) |  |
| *M. japonica* | 0.00 | (0.00 | – | 0.00) |  |
| *M. equina* | 0.00 | (0.00 | – | 0.00) |  |
| *M. nana* | 0.00 | (0.00 | – | 0.00) |  |
| *M. vespertilionis* | 0.00 | (0.00 | – | 0.00) |  |
| *M. cuniculi* | 0.00 | (0.00 | – | 0.00) |  |
| ^†^ Interquartile range (25% tile – 75% tile), ^‡^ Percentage of total fungal counts was calculated.  If *Malassezia* was detected as less than 0.01% of the total fungal population, it was noted as 0.00 in the table. | | | | | |

| **Supplementary Table 2. Relationship of six infants with IFSAT scores of 0 with four inflammatory cytokines and *Malassezia*.** | | | | | | |
| --- | --- | --- | --- | --- | --- | --- |
|  | Infants (n=6) | | | | | |
|  | A | B | C | D | E | F |
| **Four inflammatory cytokine signals**^†^ |  |  |  |  |  |  |
| IL-4 signal | Positive | Positive | Positive | Positive | Positive | Positive |
| IL-6 signal | Negative | Negative | Negative | Negative | Positive | Positive |
| IL-8 signal | Negative | Negative | Negative | Positive | Negative | Positive |
| IL-17 signal | Negative | Positive | Negative | Positive | Negative | Positive |
|  |  |  |  |  |  |  |
| ***Malassezia* genus** (%)^‡^ |  |  |  |  |  |  |
| Total *Malassezia* | 0.01 | 45.78 | 67.90 | 49.99 | 1.17 | 11.23 |
| ***Malassezia* species** (%)^‡^ |  |  |  |  |  |  |
| *M. globosa* | 0.01 | 22.30 | 22.07 | 25.86 | 0.22 | 8.91 |
| *M. restricta* | 0.00 | 1.54 | 3.44 | 12.69 | 0.71 | 1.57 |
| *M. sympodialis* | 0.00 | 3.55 | 39.16 | 0.78 | 0.06 | 0.48 |
| *M. arunalokei* | 0.00 | 0.61 | 0.43 | 9.94 | 0.09 | 0.20 |
| *M. obtusa* | 0.00 | 0.40 | 0.04 | 0.67 | 0.00 | 0.00 |
| *M. furfur* | 0.00 | 16.93 | 0.00 | 0.05 | 0.02 | 0.06 |
| ^†^ Evaluating by skin blotting methods, ^‡^ Percentage of total fungal counts was calculated. | | | | | |  |
| IL, interleukin.  If *Malassezia* was detected as less than 0.01% of the total fungal population, it was noted as 0.00 in the table. | | | | | | |
